# Accuracy of Ultrasound-Guided Ulnar Perineural Subcutaneous Aspirate for the Diagnosis and Control of Leprosy: Protocol for a Prospective Diagnostic Evaluation Study

**DOI:** 10.2196/86421

**Published:** 2026-07-29

**Authors:** Claudia Maria Escarabel, Daniel Barroso, Patricia Alves Ponte Monteiro, Lúcia Helena Gomes Fernandes, Francyne Britto Funayama Papa, Mauro da Silva Rocha, Rodolfo Rêgo Deusdará Rodrigues, Ciro Martins Gomes, Neysa Aparecida Tinoco Regattieri

**Affiliations:** 1 Faculty of Medicine Postgraduate program in medical sciences Universidade de Brasília Brasília, DF Brazil; 2 Clínica da Mama Brasilia DF Brazil

**Keywords:** leprosy, ultrasound, Mycobacterium leprae, Mycobacterium lepromatosis, molecular biology, diagnoses

## Abstract

**Background:**

Leprosy is a chronic, granulomatous, and infectious disease caused by *Mycobacterium leprae* and *Mycobacterium lepromatosis.* The diagnosis of the disease is primarily clinical based on the observation of the characteristic signs and symptoms. Laboratory tests such as bacilloscopy and skin biopsy can also be used to aid in diagnosis. Due to the lack of complementary tests with 100% sensitivity, diagnosis is often made based on the combination of clinical, laboratory, and molecular criteria. Although *M leprae* has a tropism for nerves, no validated minimally invasive procedure exists to sample nerves for this disease. The investigation of the genetic material of the etiological agents that cause leprosy through perineural collection guided by ultrasound is a promising method for improving diagnostic accuracy.

**Objective:**

This study aims to evaluate the accuracy of ultrasound-guided perineural aspirate in the diagnosis of leprosy. The World Health Organization leprosy composite reference standard will be used as the benchmark for diagnostic accuracy. We will also perform the clinical, molecular, and ultrasonographic follow-up of patients. The study will be performed at the University Hospital of Brasília, a reference center for the treatment of leprosy in the central-west region of Brazil.

**Methods:**

This will be a diagnostic study with consecutive prospective recruitment of patients referred to the leprosy outpatient clinic of University Hospital of Brasília for diagnosis and treatment. During follow-up, clinical and molecular examinations will be performed, and their results will be compared to the sonographic findings, which will be collected monthly for 1 year. Patients will be evaluated clinically, via ultrasound, and molecularly in 2 stages of leprosy diagnosis and treatment: immediately after clinical diagnosis and 1 year after the start of treatment. Subcutaneous aspiration of the ulnar nerve will be performed in the ulnar tunnel guided by ultrasound. The aspirated material will be used for identification of bacillary DNA and RNA at the time of diagnosis and 1 year after the start of treatment.

**Results:**

Grant funding for this study was received in May 2023, August 2023, and June 2026. As of October 2025, no patients have been enrolled in this study. Patient recruitment started in January 2026 and is expected to be concluded in December 2026. Collection of clinical and laboratorial data will follow the same schedule as that of recruitment. Analysis of the collected data will be conducted between February 2027 and July 2027, with results expected to be submitted to publication by August 2027.

**Conclusions:**

We believe that ultrasound-guided perineural aspirate will increase the accuracy of leprosy diagnosis and may demonstrate the viability of the bacillus before and after treatment via RNA identification.

**International Registered Report Identifier (IRRID):**

PRR1-10.2196/86421

## Introduction

### Background

Leprosy is a disease that has chronic, granulomatous, and infectious-contagious characteristics. It is caused by *Mycobacterium leprae* or *Mycobacterium lepromatosis*, which are gram-positive and acidic-resistant bacilli that have neural tropism. The disease usually affects superficial nerves of the skin and peripheral nerves. It can affect the eyes; other organs such as the liver, spleen, and testicles; and mucus and bone marrow [1]. When untreated, it evolves slowly and gradually, and its sequelae may result in physical disabilities and deformities [2-4].

The Global Leprosy Strategy 2021-2030 aims to eradicate leprosy and is part of the action plan for neglected tropical diseases [5]. One of the major challenges in the elimination and prevention of sequelae is the absence of a standard test with high sensitivity and specificity for all forms of the disease [6]. After infection, *M leprae* is preferentially concentrated in the skin and neural tissue, and neurological involvement may occur before diagnosis and constitute the only symptom of the disease [7]. Routine methods allow for the collection of cutaneous biological material for the detection of this mycobacterium. However, the collection of neural material is limited by the need to use invasive methods that can potentially lead to neurological sequelae [8].

Although special histopathological stains are routinely used to detect the mycobacterium in standard laboratories [9], molecular biology methods such as polymerase chain reaction (PCR) have greater sensitivity for diagnosis, and their incorporation into the diagnostic routine will potentially allow for earlier diagnosis with the prevention of sequelae [10-13]. The *M leprae*–specific repetitive element (RLEP) is the most commonly used target for this reaction, being also suggested to be the most sensitive and specific [13,14]. PCR also allows for measures of parasite viability that are useful in the evaluation of therapeutic response and possible drug resistance [15].

Clinically, the evaluation of possible nervous involvement is conducted via neurological examination in search of sensorimotor deficit and through palpation of nerves to evaluate nervous thickening [16]. In the appropriate clinical context, the presence of signs of peripheral neuropathy is often diagnostic for leprosy. The time when neurological deficit has already developed constitutes a very late stage for diagnosis, resulting in irreversible sequelae [17]. In addition, peripheral nerve palpation is examiner dependent and has low accuracy in the diagnosis of the disease [18].

Ultrasound has higher spatial resolution when compared with magnetic resonance imaging, which results in higher sensitivity with equivalent specificity to detect focal peripheral nerve pathology [19]. It is a noninvasive and low-cost method that provides real-time and dynamic examination, enabling the acquisition of accurate morphological information (diameter and fascicular abnormalities) without side effects for the patient. Through the Doppler mode, it is also possible to know whether there is an increase in local vascular flow indicating an inflammatory process [20,21]. Additionally, the test is also used for therapeutic procedures and for the collection of biological material in a minimally invasive way [22]. We hypothesize that minimally invasive perineural aspiration by means of high-frequency ultrasonography is feasible and that PCR performed in the aspirate will be accurate to diagnose leprosy. As the neural sequelae related to leprosy greatly impact the quality of life of patients, it is essential to improve diagnosis with a focus on neural involvement. We believe that the association of ultrasonography with minimally invasive techniques for peripheral nerve assessment is an interesting strategy to achieve this goal.

### Rationale for the Study

The diagnosis of leprosy is primarily clinical and epidemiological [2-4]. However, there are significant challenges in the current diagnostic practice. As a result of the long incubation period and changes in household contacts, complete epidemiological investigation can be difficult. Additionally, clinical examination, which includes dermato-neurological analysis identifying lesions or areas of skin with altered sensitivity and palpation to identify nerve thickening, is operator dependent and has been shown to have low agreement even among trained personnel [23].

In addition to essential clinical analysis, detection of the bacillus through microscopic examination (slit skin smear [SSS] microscopy or biopsy) confirms the diagnosis. However, the sensitivity of these tests is less than 50% due to the latency of the bacillus in nonaccessible perineural structures [24,25].

The literature shows that ultrasonography provides objective morphological data in the neural evaluation of patients with leprosy [20]. In this way, the use of ultrasound examinations aims to help in the early diagnosis of the disease, improving patients’ prognosis and reducing disease transmissibility. Additionally, it is an excellent adjunct technique to guide procedures such as perineural aspirate [26-31].

The general objective of this study is to perform clinical, molecular, and ultrasonographic follow-up of patients with leprosy treated at the University Hospital of Brasília (UHB), aiming at better accuracy of the diagnosis of leprosy. Specific objectives are to (1) evaluate the diagnostic and prognostic accuracy of clinical, molecular, and ultrasound scores in leprosy; (2) compare the analysis of the ultrasound image with the clinical evaluation of the patient with suspected leprosy; and (3) create a biorepository of DNA and complementary DNA (cDNA) samples to carry out laboratory research aimed at clarifying pathological and molecular aspects related to disease activity, with a view to develop effective early treatment for affected patients.

## Methods

### Study Design and Population Studied

This is a diagnostic study with prospective collection. Patients aged 14 years or older will be enrolled after agreeing to and signing the informed consent form. The informed consent form will be signed by the responsible adult in the case of participants under 18 years of age. This research is in line with the Declaration of Helsinki and its revision made in 2013.

The case definition will be determined by a professional external to the investigation according to the standard procedures of the leprosy outpatient clinic of the UHB, which consist of clinical history, general physical examination, and dermato-neurological examination. Case definition will follow the World Health Organization (WHO) composite reference standard for the diagnosis of leprosy [32]. Direct investigation of the bacillus will be performed via SSSs of lesions, earlobes, and elbows. After the procedure, collected material from perineural aspiration and dermal lesions will be sent to the laboratory within a maximum period of 24 hours. The results of the perineural aspiration will only be known to researchers by the end of the study. Morphological data described in the ultrasound examination will be accessed by a blinded ultrasound specialist based on deidentified ultrasound images.

Clinical and molecular examinations will be performed, and their results will be compared to the sonographic findings, which will be collected monthly for 1 year. Molecular findings will be compared to the composite reference standard. Patients will be evaluated clinically, via ultrasound, and molecularly immediately after clinical diagnosis and 1 year after the start of treatment.

### Case Definition: Composite Reference Standard

All patients with suspected leprosy referred to the outpatient clinic of the UHB will be included. Included patients will be subject to a standardized clinical evaluation and complementary evaluations. Allocation to the leprosy group will be conducted according to the WHO criteria [32], with allocation to the nonleprosy group being reserved for those patients referred by the primary care physician due to suspected leprosy who do not fulfill the WHO’s criteria [32]. Neither ultrasound nor molecular examinations performed on the perineural aspirate will be used in the allocation process. Collection of clinical data and ultrasound examinations with perineural aspirate will be performed on the same day. The diagnostic evaluation will be performed before the index test; as a result, the diagnostic classification provided by the index text will not be known by those responsible for accessing the composite reference standard. Inclusion will occur only after all standard diagnostic tests are conducted.

### Exclusion Criteria

Patients with the following comorbidities causing neuritis or neuropathy will be excluded: diabetes, hypothyroidism, HIV, trauma-related peripheral neural disease, hereditary neuropathy, autoimmune diseases, and alcoholism. Patients with relapsed leprosy who had ulnar nerve transposition and patients who, at the initial ultrasonographic examination, present anatomical alterations that may increase the risk of or bring additional risks to the subcutaneous aspirate process will also be excluded.

### Clinical Data

**Figure 1:**
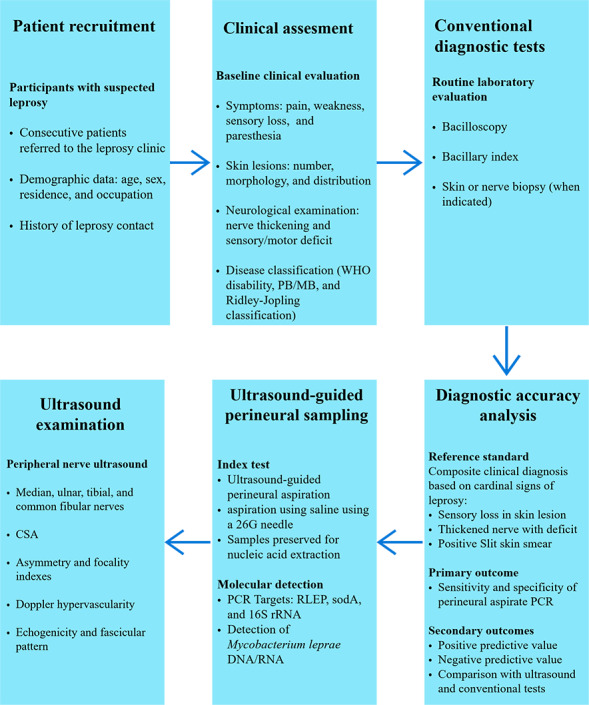
Schematic representation of the study workflow, including patient recruitment, ultrasound evaluation, perineural aspiration, and molecular testing. CSA: cross-sectional area; MB: multi-bacillary; PB: paucibacillary; PCR: polymerase chain reaction; rRNA: ribosomal RNA; WHO: World Health Organization.

The following clinical and complementary variables will be collected: age, sex, place of residence, occupation, history of household or close contact with a leprosy case, duration of symptoms, type of presenting symptoms (eg, sensory loss, paresthesia, pain, or muscle weakness), number and anatomical distribution of skin lesions, morphological characteristics of lesions (hypopigmented, erythematous, infiltrated, or nodular), presence of sensory loss within skin lesions, presence of peripheral nerve thickening on physical examination, nerves affected, sensory impairment on neurological examination, motor impairment, disability grade at diagnosis according to the WHO criteria, clinical classification of leprosy, operational classification according to WHO criteria (paucibacillary or multi-bacillary), and classification according to the Ridley-Jopling spectrum (indeterminate, tuberculoid, borderline tuberculoid, midborderline, borderline lepromatous, and lepromatous). Complementary diagnostic data will include SSS results and bacillary index when available, as well as histopathological findings from skin or nerve biopsy when performed. A standardized simplified neurological examination according to Brazil’s Ministry of Health guidelines will be conducted for all patients [33]. Table 1 aggregates all variables to be collected, and Figure 1 summarizes the study timeline.

**Table 1:** Variables to be collected.

Category	Variables
Participant characteristics	Age, sex, place of residence, occupation, and history of household or close contact with a leprosy case
Clinical and disease characteristics	Duration of symptoms, type of presenting symptoms (sensory loss, paresthesia, pain, and muscle weakness), number of skin lesions, anatomical distribution of skin lesions, morphology of lesions (hypopigmented, erythematous, infiltrated, and nodular), sensory loss within lesions, peripheral nerve thickening on physical examination, nerves affected, sensory impairment on neurological examination, motor impairment, disability grade at diagnosis (WHO^a^ criteria), clinical classification of leprosy, operational classification (paucibacillary or multi-bacillary), Ridley-Jopling classification (indeterminate, tuberculoid, borderline tuberculoid, midborderline, borderline lepromatous, and lepromatous), and simplified neurological examination according to Brazilian Ministry of Health guidelines
Conventional diagnostic tests	Slit skin smear results, bacillary index, and histopathological findings from skin or nerve biopsy (when performed)
Index test—PCR^b^ from perineural aspirate	PCR detection of Mycobacterium leprae targets: RLEP, sodA, and 16S rRNA^c^
Complementary diagnostic test—ultrasound examination	Nerves evaluated: median nerve (carpal tunnel and pronator quadratus), ulnar nerve (ulnar tunnel, immediately before the ulnar tunnel, Guyon canal, and immediately before the Guyon canal), common fibular nerve (fibular head), and tibial nerve (tarsal tunnel and 4 cm above the tarsal tunnel); CSA^d^ measurements; asymmetry indexes (change in CSA^e^ and CSA ratio); focality indexes (change in TPT^f^ and TPT ratio); intraneural or epineural Doppler signal (hypervascularity); nerve echogenicity (normal or abnormal); loss of fascicular pattern; and focal nerve thickening
Reference standard (composite clinical diagnosis)	Presence of ≥1 cardinal sign of leprosy: (1) definite sensory loss in a hypopigmented or reddish skin lesion, (2) thickened or enlarged peripheral nerve with sensory loss and/or muscle weakness, and (3) acid-fast bacilli in slit skin smear

^a^WHO: World Health Organization.

^b^PCR: polymerase chain reaction. PCR targets: RLEP, sodA, and 16S ribosomal RNA of *M leprae*.

^c^rRNA: ribosomal RNA.

^d^CSA: cross-sectional area of the nerve.

^e^Absolute difference between contralateral nerves.

^f^TPT: difference between the thickest and thinnest points along the nerve.

### Ultrasound Examination

The equipment used will be the Versana Active model (GE HealthCare) with a high-frequency multifrequency golf club transducer (18 MHz). The nerves evaluated will be (1) the median nerves at the carpal tunnel and at the level of the pronator quadratus, (2) the ulnar nerves in the ulnar tunnel and immediately before the ulnar tunnel and at the Guyon canal and immediately before the Guyon canal, (3) the common fibular nerve in the head of the fibula, and (4) the tibial nerve at the level of the tarsal tunnel and 4 cm above the tarsal tunnel. The measurements to be performed will comprise the areas obtained in the cross-section, drawing a continuous line around the nerve margin in the region of greatest thickness. All nerves in both sides of the body will be studied in the axial and transverse planes, and power Doppler will be used to detect signs of inflammation in each studied area.

The calculation of neural asymmetry or its exclusion will be determined using the following data: absolute cross-sectional area (CSA) value, asymmetry indexes (change in CSA and CSA ratio), and focality indexes (change in the difference between the thickest and thinnest points along the nerve [TPT] and TPT ratio). These are defined as follows [21]: (1) change in CSA is the absolute difference in CSA between corresponding right and left nerves at the same anatomical site, (2) CSA ratio is the ratio between the CSAs of the same nerve on opposite sides of the body—right and left, (3) change in TPT is the absolute difference between the CSA at the point of interest and a nearby reference segment of the same nerve, and (4) TPT ratio is a ratio comparing the CSA at the enlarged site with a nearby reference site. Change in TPT and TPT ratio will only be measured in the cubital and carpal tunnels.

The areas will be used to calculate asymmetry, and the value of the bilateral absolute differences of each nerve in relation to the contralateral side will be determined. Normal values for comparison were obtained from the literature.

Color Doppler will be performed with a pulse repetition frequency of 0.7 to 1.0 kHz, and intraneural or epineural color detection will be considered indicative of hypervascularity.

Nerve echogenicity will be classified as normal or abnormal. A hypoechoic pattern, one with hypoechoic areas, or even one that presents focal thickening with loss of fascicular pattern will be considered abnormal.

The examinations will be performed by 2 radiologists with prior training in sonographic neural imaging blinded to the clinical examination of the patients to ensure the impartiality of the study. A third radiologist will be responsible for calculating CSA, change in CSA, CSA ratio, change in TPT, and TPT ratio based on deidentified images collected during ultrasound examination.

### Fine Needle Aspiration

Perineural aspiration will be performed in the ultrasound room of the dermatology outpatient clinic of the UHB. Asepsis and antisepsis of the skin over the collection site will be followed via ultrasound-guided insertion of a 26G needle that is connected to a 1-mL syringe (insulin). The syringe will be prefilled with 0.6 mL of sterile saline solution, with the needle being inserted in a parallel direction to the nerve with constant negative pressure. This procedure will be repeated 3 times. The aspirate will be placed in two 1.5-mL microtubes (Axygen), with 0.3 mL of Invitrogen RNAlater solution (Thermo Fisher Scientific) added to the tube designated for RNA extraction. The samples will be stored at −80 °C until nucleic acid extraction.

### PCR Testing

#### DNA Extraction

DNA extraction will be carried out using the Invitrogen PureLink Genomic DNA Mini Kit (Thermo Fisher Scientific) according to the manufacturer’s instructions.

#### Real-Time PCR for RLEP

PCR targeting the repetitive element (RLEP) of *M leprae* will be used to detect the presence of this microorganism in the perineural aspirate. Reactions will be performed in a final volume of 30 μL containing 1X reaction buffer, 0.2 mM of deoxyribose nucleoside triphosphates, 1.5 mM of magnesium chloride, 1 U of Platinum Taq DNA Polymerase (Invitrogen), 0.2 μM of the primers 5′-CTTGCACCATTTCTGCCGCT-3′ and 5′-TGCGCTAGAAGGTTGCCGTA-3′ (Invitrogen), ultrapure water, and 50 to 100 ng of genomic DNA. Amplification will be performed using a QuantStudio 3 system (Thermo Fisher Scientific) with an initial denaturation period of 3 minutes at 94 °C followed by 15 cycles at 94 °C for 30 seconds, 56 °C for 30 seconds, and 72 °C for 30 seconds and 20 cycles at 94 °C for 1 minute, 56 °C for 30 seconds, and 76 °C for 1 minute.

#### RNA Extraction and Treatment With Deoxyribonuclease and cDNA Formation

RNA extraction, treatment with deoxyribonuclease, and cDNA formation will be carried out using validated kits according to availability of the dermatomycology laboratory of the University of Brasília Faculty of Medicine. High–molecular weight RNA will be purified using the mirVana PARIS Kit (Thermo Fisher Scientific) with 500 μL of perineural aspirate. RNA will be quantified spectrophotometrically (NanoDrop 2000 and 2000c; Thermo Fisher Scientific). A total of 500 ng of RNA will be converted to cDNA using the PureLink RNA Mini Kit (Thermo Fisher Scientific).

#### Real-Time PCR for sodA and 16S Ribosomal RNA

For these reactions, the primers targeting 16S ribosomal RNA (5′-GCATGTCTTGTGGTGGAAAGC-3′, 5’-CACCCCACCAACAAGCTGAT-3’, and probe 5′-CATCCTGCACCGCA-3′) and sodA (5’-ACCACGCCGCATATGTCA-3′, 5’-CGCGTGCCTCGTCAAGT-3’, and probe 5’-TGGCAAGCGCGTCATTGACACCT-3’) will be used. We will add 5 µl of purified *M leprae* cDNA to a mixture containing 25 µl with 2X TaqMan Universal PCR Master Mix (Thermo Fisher Scientific), 500 nM of each primer, and 100 nM of each probe. Reaction mixtures will be subjected to 50 °C for 2 minutes, 95 °C for 10 minutes, and 40 cycles at 95 °C for 15 seconds and 60 °C for 1 minute. Amplification will be performed using a QuantStudio 3 system (Thermo Fisher Scientific).

### PCR Positivity Criteria

A DNA or RNA sample will be considered positive if all the following criteria are met: threshold cycle (Ct) of 30 or less, standard amplification curve with exponential and plateau phases, and significant amplification of the positive controls with no amplification meeting the previous criteria for the negative controls. In the absence of a Ct of 30 or less or in the case of an amplification curve that does not meet the criteria above, the sample will be considered negative. Accuracy using this Ct value will be the main finding of the study, being compared with the conventional diagnostic tests and ultrasound examination. We will also construct receiver operating characteristic curves with different Ct values to evaluate whether setting different thresholds can improve the accuracy of the test compared to the preplanned value. If there is no amplification in the positive controls, or if there is amplification of the negative controls, real-time PCR for all samples in the plate will be repeated. Missing values will be dealt with using multiple imputation.

### Sample Calculation

The sample size calculation was based on the prevalence of leprosy, measured at 50% in the target population (patients with suspected leprosy referred to the UHB). Internal data also show that the sensitivity of sputum smear microscopy (standard test for detecting the bacillus) is 50%. A 20% sensitivity gain was arbitrated with perineural aspirate, resulting in a sensitivity of 70% with the proposed technique. Considering a *P* value of less than .05 and a power of more than 0.80 plus a sample increase of 10% to reduce the impact of losses, we arrived at a sample of 54 patients per group (leprosy and nonleprosy) for a total of 108 patients in the study.

The ability of ultrasonography with perineural aspirate to identify patients with leprosy diagnosed via sputum smear microscopy and skin biopsy was analyzed using the area under the curve (AUC). The range of values for the AUC varies between 0.1 and 1. The interpretations for the values are as follows: 0.5 to 0.7 is considered low, 0.7 to 0.9 is considered intermediate, and above 0.9 is considered high [34].

### Diagnostic Accuracy

The measure of diagnostic accuracy for the main objective will be the accuracy of PCR for the detection of *M leprae*. We will also calculate specificity and sensitivity of the method and of real-time PCR for the detection of *M leprae* viability. AUC will be calculated for quantitative variables obtained using ultrasound examination.

### Ethical Considerations

All patients willing to participate in this study will be required to sign an informed consent form before data collection begins. This study has been approved by the University of Brasília’s Research Ethics Committee under the number 85116624.2.0000.5558 on March 29, 2025, and adheres to the Declaration of Helsinki regarding ethical principles for human subject research. Patients will not be financially compensated.

### Study Registration

The study was registered on Plataforma Brasil [35]. It is also registered on ClinicalTrials.gov under the number NCT07515989.

### Data Collection

To safeguard sensitive information, study data will be collected and managed using the REDCap (Research Electronic Data Capture; Vanderbilt University) tool hosted at the University of Brasília. REDCap is a secure, web-based software platform designed to support data capture for research studies, providing (1) an intuitive interface for validated data entry, (2) audit trails for tracking data manipulation and export procedures, (3) automated export procedures for seamless data downloads to common statistical packages, and (4) procedures for data integration and interoperability [36,37].

## Results

Grant funding for this study was received in May 2023, August 2023, and June 2026. As of October 2025, no patients have been enrolled in this study. Participant recruitment started in January 2026 and is scheduled to end in December 2026. We expect to recruit 108 patients with suspicion of leprosy consecutively in this time frame, of whom half are expected to have the disease and half are expected to have other diagnoses, serving as nonleprosy controls. Collection of clinical and laboratorial data will follow the same schedule as that of recruitment. Data analyses are expected to begin in February 2027 and end in July 2027, with findings expected to be submitted for publication by August 2027.

## Discussion

### Expected Findings

The findings of this study are expected to support perineural aspiration as a viable diagnostic strategy for leprosy, particularly in early-stage, paucibacillary, and pure neural presentations. The application of PCR to perineural specimens will be demonstrated to have notable sensitivity and specificity in detecting *M leprae*, addressing inherent shortcomings of conventional diagnostic approaches that rely predominantly on clinical criteria and cutaneous sampling. This probable finding may transform the current management of a suspected leprosy case. Worldwide, leprosy diagnosis is based on the presence of at least one of three cardinal signs: (1) definite loss of sensation in a pale (hypopigmented) or reddish skin patch, (2) thickened or enlarged peripheral nerve with loss of sensation and/or weakness of the muscles supplied by that nerve, or (3) presence of acid-fast bacilli in an SSS [32]. Clinical examinations have limited sensitivity in multi-bacillary cases [38] and are not reliable when performed by inexperienced individuals [39,40]. These can be important factors for the re-emergence of the disease in low-prevalence areas where trained personnel and SSSs may not be widely available. SSS cytology has limited sensitivity, especially in early-stage, paucibacillary, and pure neural leprosy [32,41], being negative in approximately 70% of all cases [42]. PCR is the most accurate technique to detect *M leprae* in biological samples [13] and has been incorporated into reference laboratories of national health services in leprosy-endemic countries [43,44], with possible future application in primary care settings [14]. Thus, by ascertaining that PCR of the perineural aspirate is an accurate method to diagnose leprosy, we may be able to prevent disability by confirming early-stage, paucibacillary, and pure neural cases.

Through our study, we will be able to demonstrate a high sensitivity of the perineural aspirate in patients with paucibacillary forms of the disease. Preliminary studies in patients that have undergone nerve biopsies have shown both a high sensitivity (92% using PCR) [45] and higher bacillary index of nerve sampling compared to skin sampling [46]. The current diagnostic strategy, which samples for the microbiological diagnosis of leprosy from skin lesions, earlobes, and 3 to 6 representative skin locations [47,48], has an especially low sensitivity in paucibacillary, pure neural, and early-stage lesions [49,50], resulting in diagnostic delays, misdiagnosis [51], and mistreatment. Our potentially higher diagnostic sensitivity in paucibacillary forms is a consequence of the traditional pattern of sample collection not matching the pattern of distribution and homing of *M leprae* in the natural history of the disease. The peripheral nervous system seems to be the first to harbor this mycobacterium [52] and is the location of persistent parasites after treatment of multi-bacillary disease [53].

In this study, we will evaluate both RLEP and RNA targets (sodA and 16S). Previous studies have evaluated a variety of PCR targets to detect *M leprae* in tissues, with the most frequently used being RLEP [13]. Despite its high specificity, the use of this target can only detect 75% of patients with leprosy [13], and its positivity is not related to the viability of the microorganism [15]. We expect to obtain high assay sensitivity using these tests in parallel, as it has been proposed in other studies [14]. As *M leprae* viability is an important clinical question in leprosy, especially in terms of differentiating reaction from relapse, we will also use RNA targets. Their use better correlates with active microorganisms as this nucleic acid is less resistant to temperature and has a shorter half-life [54], being already used for viability evaluation in preliminary studies [15,41]. Thus, our proposed test is also expected to deliver information on the viability of *M leprae* in the aspirate.

The sample collection method is expected to be feasible in most primary care facilities. One of the main limitations of nerve sampling in the diagnosis of suspected leprosy has been the invasiveness of conducting nerve biopsies [55], which generally require sedation and anesthesia monitoring. However, less invasive nerve aspiration studies have been performed, being generally limited to clinically enlarged nerves [56,57]. The feasibility of the collection method proposed in our study will not be unexpected as ultrasound-guided aspiration has been used in other studies for previously inaccessible organs [58]. The use of ultrasound guidance will likely reduce the false negative rate compared to the conventional method. This will be in accordance with other studies using different samples, which have shown that guidance enhances the quality of the material obtained and improves diagnostic accuracy [20,59]. Tissue sampling via aspiration has already been shown to be feasible to collect interstitial fluids from many tissues, including orbits, eyelids, genitals, lymph nodes, and thyroid glands [58]. Similar to what we expect from our study, complications with the use of thin needles (less than 21G) are rare [58,60,61]. Using ultrasound guidance, we expect to arrive at a safe, minimally invasive collection method with high diagnostic yield suitable to be used in primary care facilities.

Although not planned in this study, the validation of this protocol may allow for the future use of the validated collection method to perform other targeted studies. In the realm of molecular evaluation, this methodology could be used to leverage specialized biomolecular markers, including circulating micro-RNAs and DNA methylation profiles, which are crucial for identifying early-stage pathologies [11,15]. The advancement of next-generation sequencing technologies and amplification techniques such as digital PCR could allow for the detailed capture of genetic and epigenetic information, providing a deeper understanding of molecular characteristics related to clinical prognosis and drug resistance [62].

One of the main limitations of this study is that aspiration will be performed in only 1 nerve. It is possible that aspiration of multiple nerves would increase the sensitivity of the method. On the other hand, this would greatly increase the time and resources needed to perform the procedure, affecting not only the feasibility of the study but also the applicability of the method in low-resource settings. Thus, collection in multiple nerves may defeat the purpose of being suitable to most primary care facilities. Additionally, the ulnar nerve is the most frequently involved in leprosy [63], and its assessment in the cubital tunnel is practical due to its proximity to clear anatomical landmarks [64].

### Conclusions

The integration of the procedures proposed in this protocol reinforces their clinical utility, emphasizing diagnostic precision and the reduction in invasive interventions. The implementation of similar methodologies has been demonstrated to result in significant reduction in the time between sample collection and definitive molecular diagnosis, as well as a decrease in operational costs compared to conventional protocols [26,28]. The proposed approach is expected to represent an important step toward personalized medicine, enhancing the capacity for early medical intervention and balancing technological innovation with cost-effectiveness and patient well-being [5].

## Data Availability

Deidentified data supporting the findings of this study will be available in the Dryad digital repository [65] following publication of the article.

## Abbreviations

AUC: area under the curve
cDNA: complementary DNA
CSA: cross-sectional area
PCR: polymerase chain reaction
REDCap: Research Electronic Data Capture
SSS: slit skin smear
TPT: difference between the thickest and thinnest points along the nerve
UHB: University Hospital of Brasília
WHO: World Health Organization
